# Agronomic and grain quality traits of oat germplasm in winter fallow fields of Sichuan, China

**DOI:** 10.3389/fpls.2025.1688513

**Published:** 2025-12-03

**Authors:** Hongying Xie, Jing Zhang, Zihan Ling, Ganqin Tang, Qiuhong Wan, Lijun Zhu, Qiujie Yan, Qinggui Wu

**Affiliations:** 1 School of Life Sciences (School of Ecological Forestry), Mianyang Normal University, Mianyang, China; 2 Forest Ecology and Conservation in the Upper Reaches of the Yangtze River Key Laboratory of Sichuan Province, Mianyang, China; 3 Engineering Research Center for Forest and Grassland Disaster Prevention and Reduction at Mianyang Normal University of Sichuan Province, Mianyang, China

**Keywords:** oat varieties, agronomic traits, nutrients, winter fallow fields, application value

## Abstract

**Introduction:**

To improve the rate of utilization of idle fields in winter and identify suitable varieties for planting, production, utilization, and oat crop quality, six oat varieties were randomly selected, and their agronomic traits were measured.

**Methods:**

Soluble sugars, crude starch, γ-aminobutyric acid, and the content of four types of phenolic nutrients were determined using anthrone, GOPOD oxidase, UV-visible spectrophotometry, Folin-Ciocalteau methods, followed by correlation analysis, respectively. The variety Challenger showed the best growth as measured by the three morphological indices (plant height, leaf width, and stem diameter).

**Results:**

For the three yield indices (fresh weight, thousand grain weight, and dry weight), the yield of Mengyan 1 and Tianyan 70 was highest and lowest, respectively. The soluble sugar content of Baylor 2 was 5.300%, which was significantly different to the other varieties, and the crude starch content of Baylor 2 was 15.044 mg/g, which was significantly higher than that of the other varieties. The GABA content of Mengyan 1 was 0.220%, which was significantly higher than that of the other varieties. The phenol content of Challenger and Qingyan 1 was significantly higher than that of the other varieties, (1.240 mg/g and 1.100 mg/g respectively), and Challenger had the highest compared with the remaining varieties. Challenger exhibited the best production performance, as determined by a comprehensive evaluation using a gray system correlation analysis, followed by Baylor 2.

**Discussion:**

The preliminary judgment is that Challenger and Baylor 2 can be promoted as the main varieties to plant in idle winter fields in the Mian-Yang area of Sichuan Province, China.

## 1 Introduction

Oats (*Avena sativa* L.) are annual herbs of the Poaceae family (Veda and Chakraborti, 2020) and are the seventh most economically important cereal after maize, rice, wheat, barley, sorghum, and millet (Boczkowska et al., 2016). The nutritional value of oats is high, and their amino acids content is rich and balanced (Sterna et al., 2016; Veda and Chakraborti, 2020). The fat content in oats is relatively high, ranking first among all grains. The components of oat fat include linoleic acid, sublinoleic acid, and monounsaturated fatty acids (Pokhrel et al., 2025; Kourimska et al., 2018). Oats are rich in polyphenols that have antioxidant functions, promote gastrointestinal digestion, lower blood pressure and lipid levels, and prevent arteriosclerosis (Kim et al., 2021; Liu et al., 2004; Li et al., 2011; Zhou et al., 2016). Biostimulants significantly influence the nutritional benefits, quality, and plant growth of crops (Jahan et al., 2024). Oats contain many important vitamins, including B1 (0.002%), B2 (0.001%), B3 (0.032%), and E (0.840%) (Rasane et al., 2015; Morales-Polanco et al., 2017).

Oats are high-quality forage crops commonly sown in fallow winter fields (Wang et al., 2020; González-García et al., 2016). They have the advantages of good adaptability, high grass yield, high digestible fiber content, and low crude fiber content, making them widely favored by livestock. They are also tolerant to drought, cold, salt, and alkali, and exhibit wide adaptability (Nan et al., 2020; Obour et al., 2019; Huang et al., 2024), enabling cultivation across extensive regions.

Winter fallow fields refer to farmlands fallow during winter after the harvest of late-season crops. According to (Wang et al., 2024), in the 2021–2022 period, ~ 260 million mu of farmland exists in southern China lying fallow for more than 100 days in winter, and ~210 million mu lying fallow for more than 120 days. Taking Sichuan Province as an example, from 2019–2022, the area of winter fallow fields in Sichuan was ~11–13 million mu. Fields that remained unused for all four years accounted for 13.4%, ~1.6 million mu; those used for two years accounted for 21%, ~2.48 million mu; and those used for three years accounted for 51%, ~6.04 million mu (Agricultural Technology Trends, 2024). Among the southern provinces and cities, including Yunnan, Fujian, and Chongqing, Sichuan has the largest area of winter fallow fields (Lin, 2023).

(Yang et al., 2023) conducted a series of experiments to select oat varieties for winter fallow fields in the Wumeng Mountains, while (Yan et al., 2023) conducted in-depth studies on functional oats for winter fallow fields in the southwestern mountains, especially in Yunnan, achieving good results. Therefore, screening excellent oat varieties for winter fallow fields in Sichuan Province can enhance the utilization of winter fallow fields to avoid the waste of farmland resources, and also alleviate the shortage of forage for livestock in spring and winter (Ren et al., 2023), and further develop the forage industry.

This study aimed to measure the agronomic traits of different oat varieties and four types of nutrients (soluble sugar, soluble starch, gamma-aminobutyric acid (GABA), and phenolics); conduct a comprehensive evaluation, comparison, and analysis of indicators, including survival, yield, and grain nutritional quality; and screen oat varieties suitable for planting, production utilization, and higher quality.

## 2 Materials and methods

### 2.1 Natural overview of the experimental site

Sichuan Province is a large agricultural province with complex terrain and diverse climates. This experiment was conducted at the Agricultural Science Institute in Mianyang City, Sichuan Province as the experimental site, located in Xinqiao Town, Youxian District, Mian Yang City. Here, hills are continuous, but slopes are gentle, and terrain is flat-bottomed with shallow hills. The thickness of the soil tillage layer is 12–18 cm, pH is 6.8-7.1, and organic matter content is 1.2-2.3%. The four seasons are distinct, with an average annual temperature of 16.4 °C. The coldest month is January, with an average temperature of 5.2 °C, and the hottest month is August, with an average temperature of 26.2 °C. Average annual precipitation is 969.6 mm, mainly concentrated in summer and autumn.

### 2.2 Experimental materials

Oat seeds were obtained from the Qinghai-Tibet Plateau Research Institute of Southwest Minzu University (located in Chengdu, Sichuan Province, China). The selected varieties included: Tianyan 70 (a high-yielding variety with wide adaptability), Qingyan 1 (bred for high cold tolerance and suitable for alpine pastoral areas), Challenger (an introduced variety known for its high forage yield and lodging resistance), Baylor 2 (characterized by high protein content and strong stress resistance), Mengyan 1 (developed for high thousand-kernel weight and grain yield), and Kona (an introduced hull-less oat variety with low fat content).

### 2.3 Planting methods

The oat varieties were sown on October 17, 2022, in a randomized complete block design for a field trial evaluating agronomic performance. Before planting, the land was plowed and leveled, and artificial furrow drilling was performed. The row length was 3 m, row spacing was 25 cm, and sowing depth was 3–4 cm. The plot area was 15 square meters (3 ×5 m), the spacing between plots was 50 cm, and protective rows were set around each plot. When sowing, compound fertilizers of nitrogen, phosphorus, and potassium were applied as base fertilizers, and the compound fertilizer was topdressed once during the tillering period. The field was maintained under rain-fed conditions. After emergence, artificial weeding was performed once and normal field management was performed throughout.

### 2.4 Main reagents and instruments

Glucose standard solution, anthrone reagent, glucose content determination kit (from Lanjiieke Technology Co., Ltd.), GABA standard, absolute ethanol (analytically pure), sodium tetraborate buffer, redistilled phenol, and sodium hypochlorite (analytically pure).

The instruments used are as follows: electronic balance (D&T, ES120D), UV spectrophotometer (Yuansi Instruments, UV-5500PC), oven (Shanghai Xinmiao Medical Equipment Manufacturing Co., Ltd., DHG-9073B5-III), multifunctional crusher (Shanghai Shuli Instrument & Meter Co., Ltd., ST-10B), water bath (Shanghai Xinmiao Medical Equipment Manufacturing Co., Ltd., HH·S21-4-S), and centrifuge (Zhongjia, SC-3610).

### 2.5 Main methods

#### 2.5.1 Measurement of agronomic traits of oat varieties

In the experimental fields in Daejeon, the five-point sampling method was used: the plot center was designated as point 1, from which two diagonals were drawn, and four points at equal distances on the diagonals were marked 2, 3, 4, and 5. From each point, two oat plants were sampled. We selected 10 mature, healthy, and undamaged plants. For each selected plant, all parameters below were measured in three technical replicates.

Plant Height: During heading stage, a tape measure was used to measure the absolute height (ground to spike apex) for all sampled individuals. Mean values were calculated (Na et al., 2018).

Leaf width: At the heading stage, the width of the fifth leaf from the tip downward was measured using a tape measure, recorded all observations, and then calculated the average.

Stem thickness: At the heading stage, the thickest part of the plant stem was measured using a caliper. All measurements were recorded, and the average value was calculated (Zhao et al., 2018).

Thousand-grain weight: Grains were harvested at full mature and air-dried. A random sample of 1000 seeds was weighed, data was recorded, and the mean was obtained.

Dry and fresh weight: At heading, fresh grass yield was weighed and then oven-dried at 105°C for 30 minutes, followed by drying at 65°C until a constant weight was achieved to determine the dry matter content. All data were recorded, and the mean value was obtained (Zhang et al., 2019). The above indicators are finally collected and analyzed by Statistical Package for the Social Sciences (SPSS).

#### 2.5.2 Determination of soluble sugar content

Soluble sugars were determined using the anthrone method in triplicate. During field trials in Daejeon, at the full maturity stage, oat straw from each variety was oven-dried and crushed using a grinder. The crushed powder was precisely weighed (50 mg) and transferred to a centrifuge tube, 5 mL of 80% ethanol was added, and incubated in an 80 °C water bath for 30 minutes. Samples were centrifuged at 4000 rpm for 10 minutes, and supernatant was collected. Extraction was repeated twice; supernatants were combined, and the final volume was adjusted to 15 mL. For color development, 1 mL of the extract was mixed with 5 mL of anthrone reagent (blank: 1 mL of 80% ethanol), boiled in a water bath for 10 min, then cooled in an ice bath. An aliquot (1 mL) was transferred to a cuvette, and the absorbance was measured at 620 nm (Xu et al., 2011).

The regression equation for the glucose standard curve was:


$$y=0.0081x-0.0251(R^{2}=0.9886)$$

#### 2.5.3 Determination of crude starch content

Starch content was determined using a glucose content assay kit in triplicate. Implemented using Glucose Oxidase Peroxidase Method (GOPOD oxidase method). During the full maturity stage of the field experiment, oat straw from each variety was collected, dried in an oven, and ground using a grinder. The powder (1 g) was accurately weighed, distilled water (10 mL) was added for grinding, and the crude grinding solution was transferred to a centrifuge tube and centrifuged at 12,000 rpm at room temperature for 10 min. The supernatant was collected for further measurements. One milliliter of the supernatant (with distilled water as the blank control) was transferred into a colorimetric cuvette, and the absorbance was measured at 520 nm.

#### 2.5.4 Determination of GABA content

Ultraviolet-visible spectrophotometry was performed in triplicate. Oat grains obtained from the field experiments were cultured in Petri dishes for 14 days, after which the seedling leaves were crushed. A 2 g sample was extracted, 12 ml of ethanol was added, and the mixture was refluxed in a 70 °C water bath for 2 h, followed by centrifugation at 4000 rpm for 10 min. Supernatants were collected for subsequent assays. An aliquot (300 µL) was mixed with 10 mL of 0.1 mol/L sodium tetraborate buffer and 400 µL of 6% phenol; 600 µL of 7.5% sodium hypochlorite solution was then added. The mixture was boiled in a water bath for 10 minutes and cooled in an ice bath for 5 minutes. Next, 2 ml of 60% ethanol was added to prepare the test solution. An aliquot (1 mL) of the test solution (blank: identical reagents without supernatant) was transferred to a cuvette, and absorbance was measured at 645 nm (Chen et al., 2006).

The regression equation for the GABA standard curve was:


$$y=0.0647x+0.0095(R^{2}=0.9891)$$

#### 2.5.5 Determination of phenolic substances

Total phenolics were determined by the Folin-Ciocalteu method (Peterson et al., 2001) in three replicates. Oat grains from field trials were cultured in Petri dishes for 14 d; seedling leaves were then ground. An aliquot of the homogenate (0.1– 0.5 g) was placed in a centrifuge tube, extracted with 5–10 mL of 70% ethanol or methanol with cooling on ice, followed by centrifugation at 8000 rpm for 10 min to collect the supernatant. The supernatant was transferred to a 50 mL volumetric flask, and its residue was repeatedly washed with the extraction solvent, and combined extracts were finally brought to volume with solvent and mixed to yield the test solution. For color development, 0.5 mL of the test solution (blank: the same volume of ethanol/methanol) was mixed with 0.5 mL distilled water and 0.5 mL of Folin phenol reagent, allowed to stand for 8 min, then 2 mL 7.5% sodium carbonate solution was added. After mixing, the reaction was kept in the dark for 60 min, and absorbance was measured at 765 nm.

The regression equation of gallic acid standard curve was:


$$y=0.0011x+0.0006(R^{2}=0.9972)$$

#### 2.5.6 Statistical analysis

The data were organized using Microsoft Excel and presented as mean ± standard deviation. Statistical analysis was performed using SPSS 27.0, with a significance level set at 0.05. Graphs were created using Origin software.

To examine the effects of different oat varieties (a categorical independent variable) on various measured indicators (continuous dependent variables), one-way analysis of variance (ANOVA) was used. If the ANOVA results showed significant differences between groups (*p* < 0.05), Duncan’s multiple range test was further applied for *post hoc* pairwise comparisons to identify which specific treatment groups differed significantly. If the *p*> 0.05, it was concluded that the overall group means showed no significant differences, and no further multiple comparisons were conducted.

Correlations were analyzed using bivariate correlation analysis in SPSS 27.0, with results presented in matrix form. This approach allows obtaining the correlation coefficients and significance levels (*p*-values) for each pair of variables, enabling a systematic assessment of the strength, direction, and statistical significance of relationships between variables.

#### 2.5.7 Grey correlation analysis

A comprehensive evaluation of oat varieties was conducted using the grey relational analysis (GRA)method, initially screening for varieties with overall superior traits suitable for cultivation in saline-alkali soils. GRA quantifies between a reference (parent) sequence and one or more comparison (feature) sequences by calculating gray relational coefficients between data, thereby supporting multi-criteria decision-making.

## 3 Results and discussion

This study systematically evaluated the performance of six oat varieties in terms of key agronomic traits and nutritional qualities. The following results detail the differences among the varieties in terms of morphology, yield, nutritional components, and their interrelationships:

### 3.1 Agronomic traits

The mature plant height of Tianyan 70 was 113 cm, which was significantly greater than that of the other varieties (**Table 1**). Challenger ranked second at 101.000cm, while Mengyan 1 (84.200 cm), Baylor 2 (84.300 cm), and Kona (85.000 cm) comprised significantly lower plant heights, classifying them as short-stature types. The leaf width of Challenger was 3.140 cm, suggesting a potentially stronger photosynthetic capacity. Kona had the narrowest leaves (2.240 cm), showing a significant difference from Challenger. The stem diameters of Challenger and Mengyan 1 were 8.020 mm and 5.650 mm, which were significantly larger and smaller than those of the other varieties, respectively. Among the three key morphological indicators (plant height, leaf width, and stem diameter), Challenger demonstrated the most robust and ideal aboveground growth, indicating its potential for high yield and lodging resistance.

**Table 1:** Morphological indices of the different oat varieties.

Variety	Plant height (cm)	Leaf width (cm)	Stem diameter (mm)
Mengyan 1	84.200 ± 2.200d	2.620 ± 0.480ab	5.650 ± 0.250d
Qingyan 1	91.500 ± 1.500c	2.840 ± 0.200ab	7.320 ± 0.120b
Baylor 2	84.300 ± 4.300d	2.680 ± 0.130ab	7.620 ± 0.190b
Challenger	101.000 ± 1.000b	3.140 ± 0.160a	8.020 ± 0.200a
Tianyan 70	113.000 ± 4.000a	2.740 ± 0.440ab	6.880 ± 0.280c
Kona	85.000 ± 4.000d	2.240 ± 0.360b	5.860 ± 0.240d

The three morphological indices–plant height, leaf width, and stem thickness–of the different oat varieties were compared. Different letters in the same column indicate significant differences (*p*<0.05).

The fresh weight of Qingyan 1 is 0.222 kg, which is the highest among all varieties and shows a significant difference from Baylor 2 (0.132 kg) and Challenger (0.153 kg). (**Table 2**). The fresh weight of Tianyan 70 was 0.082 kg, which was significantly different from that of the other varieties, in contrast to its taller plant height, suggesting a weaker biomass accumulation capability. Mengyan 1 possessed the highest thousand-grain weight (3.770 g), significantly superior to that of other varieties except Baylor 2, directly relating to its plump kernels and appearance quality. Tianyan 70 had the lowest thousand-grain weight (2.400 g). Although the dry weight did not reach statistical significance among varieties, Qingyan 1 (0.061 kg) and Tianyan 70 (0.066 kg) showed a trend towards higher values, while Kona (0.042 kg) comprised a relatively lower dry weight.

**Table 2:** Production performance of different oat varieties.

Variety	Fresh weight (kg)	Thousand grain weight (g)	Dry weight (kg)
Mengyan 1	0.121 ± 0.024bc	3.770 ± 0.470a	0.043 ± 0.022a
Qingyan 1	0.222 ± 0.012a	2.510 ± 0.190cd	0.061 ± 0.008a
Baylor 2	0.132 ± 0.020b	3.280 ± 0.280ab	0.043 ± 0.013a
Challenger	0.153 ± 0.033b	3.040 ± 0.150bc	0.058 ± 0.009a
Tianyan 70	0.082 ± 0.017d	2.400 ± 0.400d	0.066 ± 0.027a
Kona	0.093 ± 0.006cd	3.050 ± 0.150bc	0.042 ± 0.010a

The three production performances of different varieties of oats were compared in terms of fresh, 1000-grain, and dry weights. Different letters in the same column indicate significant differences (*p*<0.05).

### 3.2 Crude starch content

The crude starch content of Baylor 2 was 15.044 mg/g, which was significantly higher than that of the other varieties (**Table 3**), making it an ideal candidate for high-energy feeds. Except for Qingyan 1 and Tianyan 70, the crude starch contents of all varieties were significantly different (*p*<0.05). Qingyan 1 (12.389 mg/g) and Tianyan 70 (12.168 mg/g) belonged to the medium-high starch content group. The crude starch content of Kona was 4.646 mg/g, which was significantly lower than that of the other varieties.

**Table 3:** Crude starch content of different oat varieties.

Variety	Glucose content (mg/g)	Crude starch content (mg/g)
Mengyan 1	7.965 ± 0.165d	8.850 ± 0.010d
Qingyan 1	11.150 ± 0.026b	12.389 ± 0.089b
Baylor 2	13.540 ± 0.100a	15.044 ± 0.044a
Challenger	8.761 ± 0.210c	9.735 ± 0.035c
Tianyan 70	10.951 ± 0.100b	12.168 ± 0.168b
Kona	4.181 ± 0.100e	4.646 ± 0.046e

The light absorption value, glucose content, and coarse starch content of different varieties of oats were compared. Different letters in the same column indicate significant differences (*p*<0.05).

### 3.3 Soluble sugar content

**Figure 1:**
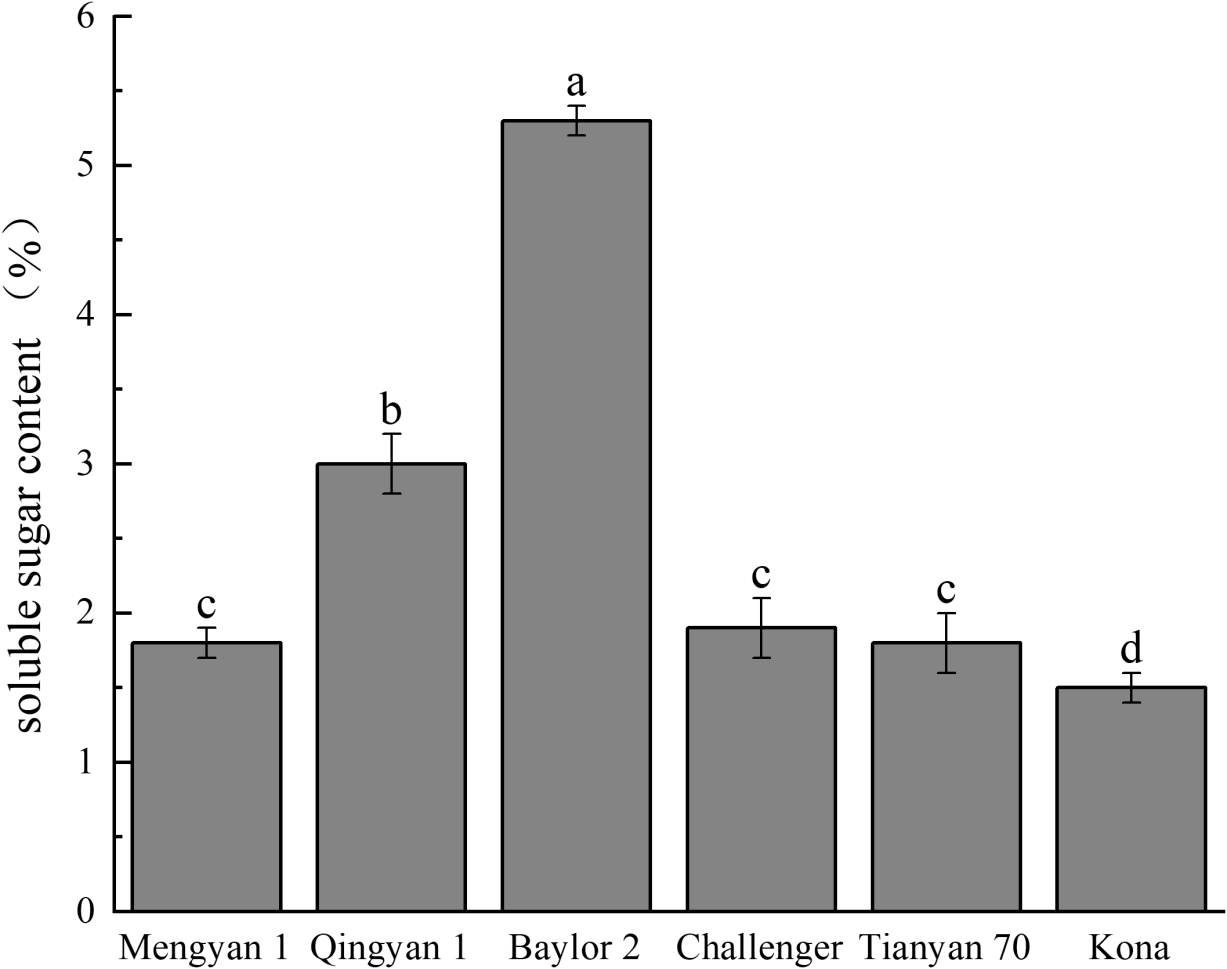
Soluble sugar content in different oat varieties. Different lowercase letters within a column indicate significant differences (*p*<0.05).

The variation trend of soluble sugar content was similar to that of crude starch (**Figure 1**). Among the oat varieties, the soluble sugar content of Baylor 2 was 5.300%, significantly higher than that of the other varieties, suggesting potentially better palatability. The soluble sugar content of kona was 1.500%, significantly lower than that of the other varieties. Significant differences existed in soluble sugar content between Baylor 2 and Qingyan 1 (*p*<0.05), and between Qingyan 1 and Mengyan 1(*p*<0.05).

### 3.4 GABA and phenolic content

**Figure 2:**
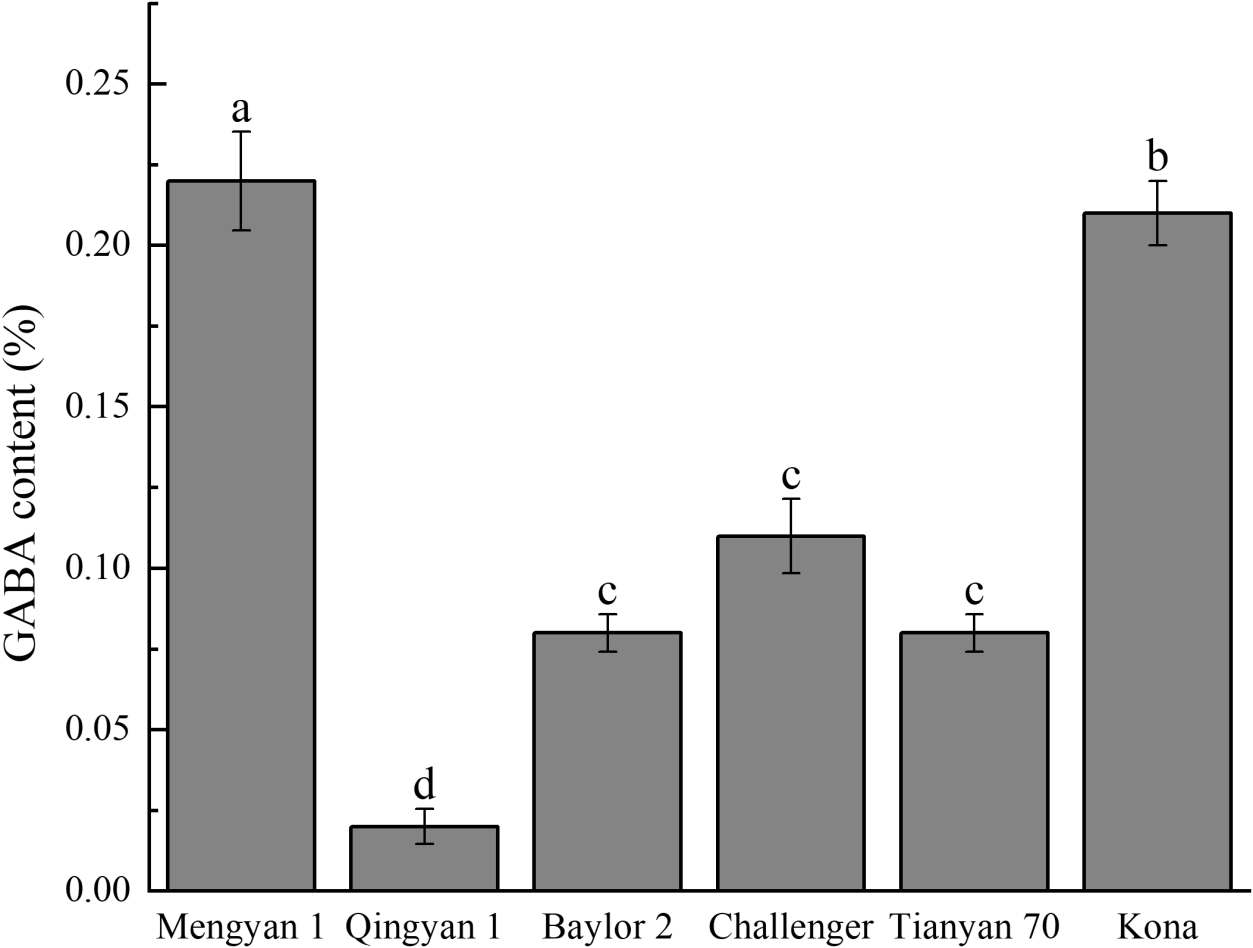
GABA content in different oat varieties. Different lowercase letters within a column indicate significant differences (*p*<0.05).

GABA is an important functional amino acid, and its content varies markedly among varieties (**Figure 2**). The GABA contents of Mengyan 1 (0.220%) and Qingyan 1 (0.020%) were significantly higher and lower than those of the other varieties, respectively. Significant differences existed in GABA content between Kona and Baylor 2, and between Challenger and Qingyan 1 (*p*<0.05).

**Figure 3:**
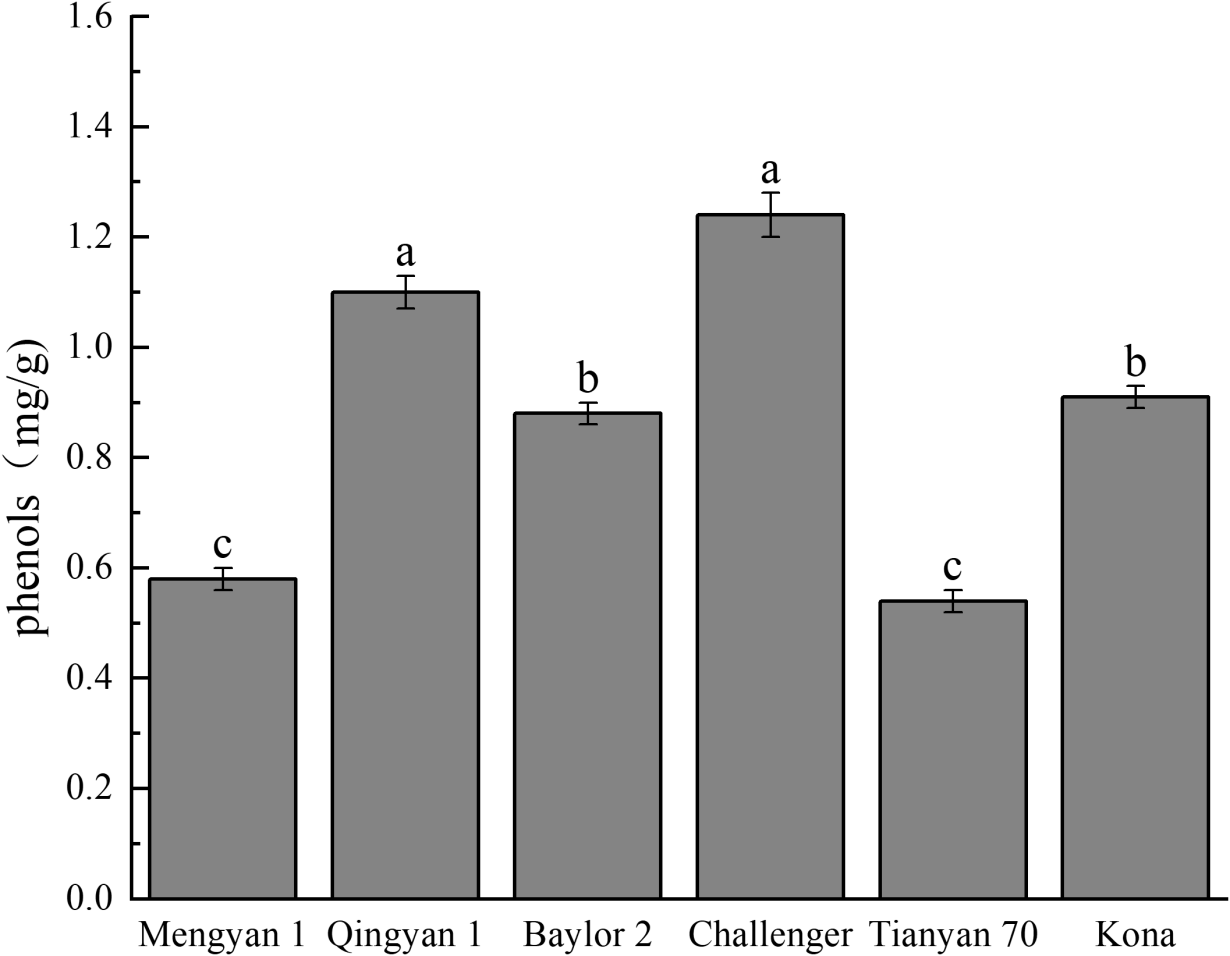
Phenolic content in different oat varieties. Different lowercase letters within a column indicate significant differences (*p*<0.05).

Phenolic compounds were found to be the major antioxidant components (**Figure 3**). The challenger had the highest total phenolic content (1.240 mg/g), indicating the strongest antioxidant potential. Qingyan 1 (1.100 mg/g) followed the closest trend. The phenolic content of these two varieties was significantly higher (*p*<0.05) than that of Baylor 2, Kona, Mengyan 1, and Tianyan 70.

### 3.5 Correlation analysis of production performance and quality characteristics

Plant height was negatively correlated with the quality characteristic indices, except for crude starch, positively correlated with production performance indices, except for fresh weight and thousand grain weight, and significantly positively correlated with dry weight (*p*<0.05) (**Table 4**). Leaf width was positively correlated with quality characteristic indices, except for GABA, as well as production performance indices, except for thousand grain weight. There was a positive correlation between stem diameter and quality characteristic indices, except for GABA, and with production performance indices, except for thousand grain weight. Fresh weight was negatively correlated with thousand grain weight, but positively correlated with dry weight and quality characteristic indices, except for GABA. Thousand grain weight was significantly negatively correlated with dry weight (*p*<0.05) but positively correlated with the quality characteristic indices of soluble sugar and GABA, and negatively correlated with crude starch and phenol. Dry weight was positively correlated with crude starch and phenol, and negatively correlated with the other two quality characteristic indices. Soluble sugar was negatively correlated with GABA but positively correlated with the other two quality characteristic indexes. Crude starch was negatively correlated with GABA and phenol content. GABA and phenol levels were negatively correlated.

**Table 4:** Correlation analysis between production performance and quality characteristics.

Variable	Plant height	Leaf width	Stem diameter	Fresh weight	Thousand grain weight	Dry weight	Soluble sugar	Crude starch	GABA	Phenol
Plant height	1	–	–	–	–	–	–	–	–	–
Leaf width	0.504	1	–	–	–	–	–	–	–	–
Stem diameter	0.375	0.791	1	–	–	–	–	–	–	–
Fresh weight	-0.190	0.521	0.484	1	–	–	–	–	–	–
Thousand grain weight	-0.697	-0.223	-0.397	-0.187	1	–	–	–	–	–
Dry weight	0.881*	0.631	0.494	0.268	-0.832*	1	–	–	–	–
Soluble sugar	-0.353	0.111	0.504	0.313	0.094	-0.231	1	–	–	–
Crude starch	0.220	0.517	0.658	0.351	-0.248	0.354	0.770	1	–	–
GABA	-0.428	-0.589	-0.789	-0.593	0.714	-0.697	-0.504	-0.788	1	–
Phenol	-0.114	0.456	0.637	0.667	-0.169	0.120	0.157	-0.022	-0.358	1

Correlation analysis between the production performance and quality characteristics of the different oat varieties was performed. * indicates a significant association (*p* < 0.05).

### 3.6 Grey system correlation degree method comprehensive evaluation

To overcome the limitations of evaluating single traits, we used the GRA method to comprehensively evaluate all 10 agronomic and quality traits (**Table 5**). Based on the weighted relational degree, the overall performance of the six varieties, from best to worst, were: Challenger (0.802) > Baylor 2 (0.777) > Qingyan 1 (0.735) > Tianyan 70 (0.732) > Mengyan 1 (0.728) > Kona (0.718).

**Table 5:** Comprehensive evaluation of production performance of different oat varieties.

Variety	Weighted correlation coefficient	Rank
Mengyan 1	0.728	5
Qingyan 1	0.735	3
Baylor 2	0.777	2
Challenger	0.802	1
Tianyan 70	0.732	4
Kona	0.718	6

Gray correlation analyses of plant height, stem thickness, leaf width, fresh weight, 1000-grain weight, dry weight, soluble sugar, coarse starch, γ-aminobutyric acid, and phenolic compounds were performed.

This result indicates that Challenger had the most balanced and excellent traits, particularly with respect to morphological development and antioxidant accumulation. Baylor 2 followed closely, with its advantages mainly reflected in its high sugar and starch contents, making it an ideal choice for high-energy feed.

## 4 Discussion

Oats are widely cultivated worldwide as cereal and forage crops (Kapoor and Batra, 2016), and have specific applications in feeding (Wang et al., 2024), food processing (Kulthe et al., 2014), and healthy diets (Aparicio-García et al., 2021; Alemayehu et al., 2023; Rafique et al., 2022). The agronomic traits and nutrient content of six oat varieties were compared in this study to identify those oat varieties with the highest yields and which are most suitable for planting.

Among agronomic traits, plant height and 1000-grain weight have been most strongly correlated with oat grain yield and hay yield (Luo et al., 2024), and (Shah et al., 2016) showed that plant height and stem thickness can be used as indicators of forage yield and lodging resistance (Awais et al., 2015; Argenta et al., 2022). In the present study, among the three morphological indices of plant height, stem diameter, and leaf width, the oat variety Challenger showed the best growth, and it was inferred that Challenger had the highest grain yield and strongest lodging resistance. Challenger’s plant height was 101.000 cm, which is higher than the plant height for this variety (89.700 cm) in a study comparing the adaptability of 20 oat varieties in Yinchuan irrigation area (Ke, 2023). The 1000-grain weight of Mengyan 1 was the highest of the six oat varieties in our study, in alignment with the findings of (Sun et al., 2014) who studied various agronomic traits and the yield of nine cultivated oat varieties. In the study by (Sun et al., 2014) there was a significant positive correlation between oat yield and 1000-grain weight indicating that the oat hay yield obtained by the subsequent planting of Mengyan 1 would be high. In the present study, the soluble sugar content of Baylor 2 was 5.300%, which was significantly higher than that of the other varieties. (Zhao et al., 2023) compared and evaluated the drought resistance of 15 forage oat germplasm varieties during the seedling stage and concluded that soluble sugar content increased significantly under drought stress. The soluble sugar content of Baylor 2 was highest, and this variety had the strongest drought resistance as indicated by the crude starch content (15.044 mg/g), which was highest. Studies have shown that the content of crude starch is positively correlated with Glycemic Index (GI), and the GI of the oat variety Kona is low (Brummer et al., 2012; Wang and Ellis, 2014; Othman et al., 2011). A low GI, which can be created in the processing of multigrain steamed bread, can help glucose to be slowly released into the blood during absorption, avoiding sharp fluctuations in blood sugar, and therefore be healthy for the human body (Long, 2021; Amerizadeh et al., 2023). The GABA content of Mengyan 1 was 0.220% in the present study, which was the highest among all six oat varieties. GABA has been reported to play a role in neural regulation and has been associated with sleep quality in some studies (Hepsomali et al., 2020; Fan et al., 2025). Therefore, the high GABA content in Mengyan 1 suggests its potential use in functional foods aimed at promoting relaxation or sleep support. The main antioxidant component in oats is phenol (Chen et al., 2015; Varga et al., 2018), and the phenol content of Challenger was 1.240 mg/g, which was significantly higher than that of the other varieties. The stronger antioxidant properties of Challenger and Qingyan 1 support use of these varieties in antioxidant products (Shah et al., 2016; Minatel et al., 2017).

As plant proteins are healthier, safer, and more environmentally-friendly than animal proteins, they are gradually replacing animal proteins (Van Zanten et al., 2018; Heusala et al., 2020; Boukid, 2021). Compared with other grains, oats have a high protein content at between 12% and 15% (Beloshapka et al., 2016; Mäkinen, O. E. et al., 2017; Mel and Malalgoda, 2022). Compared with protein from legumes and wheat, oat protein is produced at a lower cost and has a lower allergen content (Dhanjal et al., 2016; Spaen and Silva, 2021). Crude protein can be used as an index of forage quality, however protein content was not measured in this study. It is therefore recommended that the protein composition of the two dominant oat varieties identified in this study be studied further, to provide a stronger basis for oat utilization.

From the perspective of winter fallow field utilization, the core goal of selecting oat varieties is to achieve the highest biomass (forage yield) and excellent feed quality within a limited growing season. Herein, “fresh weight” and “dry weight” are key yield traits directly reflecting biomass accumulation, determining yield per unit area, and directly relating to economic benefits for farmers. In terms of quality, the content of “soluble sugars” is an important factor affecting forage palatability and animal energy intake; “crude starch” provides digestible energy; and “phenolic compounds” relate to the antioxidant health of animals. These traits often involve trade-offs; for example, high-biomass varieties may have lower nutrient concentrations (Shabana et al., 1980).

Our comprehensive evaluation results indicate that Challenger and Baylor 2 perform best in balancing these tradeoffs. Challenger exhibits a “balanced high-yield” pattern: it performs best in key morphological traits such as plant height and stem thickness, providing a structural foundation for high yield, which ultimately translates into considerable fresh and dry weights. Simultaneously, it does not sacrifice quality because its phenolic compound content is the highest among all varieties, giving it excellent antioxidant potential. This implies that planting Challenger can achieve a high yield while providing healthier forage.

Level, Baylor 2 represents a “quality-oriented” pattern: its biomass is not the highest, but still at an above-average level. Its outstanding feature is its high content of soluble sugars and crude starch, which makes it an excellent energy feed source. For livestock requiring rapid weight gain from late winter to early spring or high-energy feed, Baylor 2 has irreplaceable value.

Therefore, for the utilization of winter fallow fields in Sichuan, if the goal is to obtain the maximum amount of general-purpose forage, Challenger is the preferred variety. If the feeding strategy focuses more on supplementing high-energy feed, Baylor 2 is more advantageous. These two varieties achieved a good balance between yield and quality through different strategies, thus meeting the diverse utilization requirements of winter fallow fields.

## 5 Conclusions

In summary, the comparative analysis of agronomic traits and the content of four nutrients in six oat varieties in this study showed that Challenger and Baylor 2 were the top performing varieties. Therefore, these two varieties are recommended as suitable for planting in fallow winter fields and can be used for animal feed and human food processing. The findings provide a scientific basis for selecting oat varieties that maximize biomass and nutritional quality in fallow field systems, supporting future breeding programs and sustainable agricultural practices in similar ecological regions.

## Data Availability

The raw data supporting the conclusions of this article will be made available by the authors, without undue reservation.

## References

[B1] Agricultural Technology Trends (2024). Available online at: https://www.Chinawestagr.com/homepage/showcontent.asp?id=58739 (Accessed December 17, 2024).

[B2] Alemayehu G. F. Forsido S. F. Tola Y. B. Amare E. (2023). Nutritional and phytochemical composition and associated health benefits of oat (Avena sativa) grains and oat-based fermented food products. Sci. World J. 16, 2730175. doi: 10.1155/2023/2730175, PMID: 37492342 PMC10365923

[B3] Amerizadeh A. Ghaheh H. S. Vaseghi G. Farajzadegan Z. Asgary S. (2023). Effect of oat (Avena Sativa L.) Consumption on lipid profile with focus on triglycerides and high-density lipoprotein cholesterol (Hdl-C): an updated systematic review. Curr. Probl. Cardiol. 48, 101153. doi: 10.1016/j.cpcardiol.2022.101153, PMID: 35192870

[B4] Aparicio-García N. Martínez-Villaluenga C. Frias J. Crespo Perez L. Fernández C. F. Alba C. . (2021). A novel sprouted oat fermented beverage: evaluation of safety and health benefits for celiac individuals. Nutrients 13, 2522. doi: 10.3390/nu13082522, PMID: 34444682 PMC8401588

[B5] Argenta J. Pacheco M. T. de Araujo Mariath J. E. Federizzi L. C . (2022). Morphological, anatomical, and chemical characteristics associated with lodging resistance in Avena sativa. Euphytica 218, 22. doi: 10.1007/s10681-022-02971-8

[B6] Awais S. M. Shah S. H. Akhtar L. H. Minhas R. Bukhari M. S. Ghani A. R. . (2015). Evaluation of different oat (Avena sativa L.) varieties for forage yield and related characteristics. Sci. Lett. 3, 13–16.

[B7] Beloshapka A. N. Buff P. R. Fahey G. C. Swanson K. S. (2016). Compositional analysis of whole grains, processed grains, grain co-products, and other carbohydrate sources with applicability to pet animal nutrition. Foods 5, 23. doi: 10.3390/foods5020023, PMID: 28231117 PMC5302337

[B8] Boczkowska M. Podyma W. Łapiński B. (2016). “ Oat,” in Genetic and Genomic Resources for Grain Cereals Improvement. Eds. Singh M. Hari. D. (USA: Academic Press), 159–225.

[B9] Boukid F. (2021). Plant-based meat analogues: from niche to mainstream. Eur. Food Res. Technol. 247, 297–308. doi: 10.1007/s00217-020-03630-9

[B10] Brummer Y. Ruedi D. Thomas W. Susan M. T. (2012). Glycemic response to extruded oat bran cereals processed to vary in molecular weight. Cereal Chem. 89, 255–261. doi: 10.1094/CCHEM-03-12-0031-R

[B11] Chen D. F. Shi J. L. Hu X. Z. Du. S. K. (2015). Alpha-amylase treatment increases extractable phenolics and antioxidant capacity of oat (Avena nuda L.) flour. J. Cereal Sci. 65, 60–66. doi: 10.1016/j.jcs.2015.06.019

[B12] Chen E. C. Zhang M. W. Peng C. Y. Zhang R. F. Zhang Y. Chi J. W. (2006). Rapid determination of γ-aminobutyric acid content in brown rice by colorimetric method. J. Chin. Cereals Oils 01), 125–128. doi: 10.3321/j.issn:1003-0174200601029

[B13] Dhanjal N. I. Sharma S. Prakash N. T. (2016). Quantification and *in vitro* bioaccessibility of selenium from Osborne fractions of selenium-rich cereal grains. Cereal Chem. 93, 339–343. doi: 10.1094/CCHEM-10-15-0199-R

[B14] Fan R. Jia Y. Chen Z. Li S. Qi B. Ma A. (2025). Foods for sleep improvement: a review of the potential and mechanisms involved. Foods 14, 1080. doi: 10.3390/foods14071080, PMID: 40238208 PMC11988850

[B15] González-García S. Baucells F. Feijoo G. Moreira M. T. (2016). Environmental performance of sorghum, barley and oat silage production for livestock feed using life cycle assessment. Resour. Conserv. Recycl. 111, 28–41. doi: 10.1016/j.resconrec.2016.04.002

[B16] Hepsomali P. Groeger J. A. Nishihira J. Scholey A. (2020). Effects of oral Gamma-Aminobutyric Acid (GABA) Administration on stress and sleep in humans: A Systematic Review. Front. Neurosci. 14. doi: 10.3389/fnins.2020.00923, PMID: 33041752 PMC7527439

[B17] Heusala H. Sinkko T. Mogensen L. Knudsen M. T. (2020). Carbon footprint and land use of food products containing oat protein concentrate. J. Clean. Prod. 276, 122938. doi: 10.1016/j.jclepro.2020.122938

[B18] Huang H. Wang X. Li J. Gao Y. Yang Y. Wang R. . (2024). Trends and directions in oats research under drought and salt stresses: a bibliometric analysis (1993–2023). Plants 13, 1902. doi: 10.3390/plants13141902, PMID: 39065428 PMC11279746

[B19] Jahan M. S. Hasan M. M. Rahman M. A. (2024). Editorial: hormones and biostimulants in plants: physiological and molecular insights on plant stress responses. Front. Plant Science. 15. doi: 10.3389/fpls.2024.1413659, PMID: 38812736 PMC11133861

[B20] Kapoor R. Batra C. (2016). “ Oats,” in Broadening the Genetic Base of Grain Cereals. Eds. Singh M. Kumar S. ( Springer, New Delhi).

[B21] Ke Q. (2023) in Adaptability evaluation of 20 oat varieties in Yinchuan irrigation district ( Ningxia University).

[B22] Kim I. S. Hwang C. W. Yang W. S. Kim C. H. (2021). Multiple antioxidative and bioactive molecules of oats (Avena Sativa L.) in human health. Antioxidants 10, 1454. doi: 10.3390/antiox10091454, PMID: 34573086 PMC8471765

[B23] Kourimska L. Sabolova M. Horcicka P. Rys S. Bozik M. (2018). Lipid content, fatty acid profile, and nutritional value of new oat cultivars. J. Cereal Sci. 84, 44–48. doi: 10.1016/j.jcs.2018.09.012

[B24] Kulthe A. A. Pawar V. D. Kotecha P. M. Chavan U. D. Bansode V. V. (2014). Development of high protein and low calorie cookies. J. Food Sci. Technol. 51, 153–157. doi: 10.1007/s13197-011-0465-2, PMID: 24426062 PMC3857413

[B25] Li Y. Luo Y. C. Chen G. Xiao Z. L. Wang Q. Zhao L. . (2011). Comparison of protective effects between oat β-glucan and phenol-rich extracts in hyperlipidemic ICR mice. J. Food Drug Anal. 19, 49–57. doi: 10.38212/2224-6614.2190

[B26] Lin Z. R. (2023) in Research on Information Extraction and Development Management Strategies for Winter Fallow Fields in Ten Southern Provinces (Tianjin: Tianjin University of Technology). doi: 10.27357/d.cnki.gtgyu.2022.000788

[B27] Liu L. Zubik L. Collins F. W. Marko M. Meydani M. (2004). The antiatherogenic potential of oat phenolic compounds. Atherosclerosis 175, 39–49. doi: 10.1016/j.atherosclerosis.2004.01.044, PMID: 15186945

[B28] Long J. L. (2021) in Study on the development and quality characteristics of multi-grain steamed buns with low GI ( Hebei University of Economics and Business).

[B29] Luo J. K. Zhang K. H. Wang Z. Y. Pingzhen Z. Ming N. (2024). Study on production performance of 18 Oat varieties in the irrigation area along the Yellow River in Baiyin City. Crops 41, 93–100. doi: 10.16035/j.issn.1001-7283.2025.02.013

[B30] Mäkinen O. Sozer N. Ercili-Cura D. Poutanen K. (2017). “ Chapter 6 - Protein from oat: Structure, processes, functionality, and nutrition,” in Sustainable Protein Sources. Eds. Nadathur S. R. Janitha P. D. Wanasundara L. S. (USA: Academic Press), 105–119.

[B31] Mel R. Malalgoda M. (2022). Oat protein as a novel protein ingredient: Structure, functionality, and factors impacting utilization. Cereal Chem. 99, 21–36. doi: 10.1002/cche.10488

[B32] Minatel I. O. Borges C. V. Ferreira M. I. Gomez H. A. G. Chen C.-Y. O. Lima G. P. P. (2017). Phenolic compounds: functional properties, impact of processing and bioavailability. Tech. doi: 10.5772/66368

[B33] Morales-Polanco E. Campos-Vega R. Gaytán-Martínez M. Enriquez L. G. Loarca-Piña. G. (2017). Functional and textural properties of a dehulled oat (Avena sativa L) and pea (Pisum sativum) protein isolate cracker. LWT 86, 418–423. doi: 10.1016/j.lwt.2017.08.015

[B34] Na R. S. Liang Q. W. Yang X. F. Xiang K. F. Zhang Q. Q. Guo Z. Z. Q. . (2018). Evaluation of the production performance of 13 oat varieties in the Horqin sandy land. Heilongjiang Anim. Husbandry Veterinary 17), 136–141. doi: 10.13881/j.cnki.hljxmsy.2018.02.0158

[B35] Nan M. Jing F. Bian F. Ren S. L. Liu Y. M . (2020). Comparison of performance and feeding value of six naked oat varieties in yintao irrigation district, central gansu province. J. Grassl. Sci. 28, 1635–1642. doi: 10.11733/j.issn.1007-0435.2020.06.017

[B36] Obour A. K. Holman J. D. Schlegel A. J. (2019). Seeding rate and nitrogen application effects on oat forage yield and nutritive value. J. Pl. Nutr. 42, 1452–1460. doi: 10.1080/01904167.2019.1617311

[B37] Othman R. A. Moghadasian M. H. Jones P. J. (2011). Cholesterol-lowering effects of oat β-glucan. Nutr. Rev. 69, 299–309. doi: 10.1111/j.1753-4887.2011.00401, PMID: 21631511

[B38] Peterson D. M. Emmons C. L. Hibbs A. H. (2001). Phenolic antioxidants and antioxidant activity in pearling fractions of oat groats. J. Cereal Sci. 33, 97–103. doi: 10.1006/jcrs.2000.0347

[B39] Pokhrel K. Kouřimská L. Bhujel N. K. Parajuli R. Božik M . (2025). Comparison of the lipid content and fatty acid composition of two hulled oats and their hull with naked and dehulled oats varieties. Czech J. Food Sci. 43, 152–159. doi: 10.17221/172/2024-CJFS

[B40] Rafique H. Dong R. Wang X. Alim A. Aadil R. M. Li L. . (2022). Dietary-nutraceutical properties of oat protein and peptides. Front. Nutr. 9. doi: 10.3389/fnut.2022.950400, PMID: 35866075 PMC9294724

[B41] Rasane P. Jha A. Sabikhi L. Kumar A. Unnikrishnan V. S. (2015). Nutritional advantages of oats and opportunities for its processing as value added foods - a review. J. Food Sci. Tech. 52, 662–675. doi: 10.1007/s13197-013-1072-1, PMID: 25694675 PMC4325078

[B42] Ren X. C. Gan W. Ren X. S. Jiang X. B. Ma B. R. Yu X. . (2023). Comparative study on performance and nutritional quality of different oat varieties during winter leisure in eastern Sichuan. Feed Study. 46, 139–143. doi: 10.13557/j.cnki.issn1002-2813.2023.11.029

[B43] Shabana R. Bailey T. Frey K. J. (1980). Production traits of oats selected under low, medium, and high productivity1. Crop Sci. 20, 739–744. doi: 10.2135/cropsci1980.0011183X002000060015x

[B44] Shah A. Masoodi F. A. Gani A. Ashwar B. A. (2016). Newly released oat varieties of himalayan region–techno-functional, rheological, and nutraceutical properties of flour. LWT 70, 111–118. doi: 10.1016/j.lwt.2016.02.033

[B45] Spaen J. Silva J. V. C. (2021). Oat proteins: review of extraction methods and techno-functionality for liquid and semi-solid applications. LWT 147. doi: 10.1016/j.lwt.2021.111478

[B46] Sterna V. Zute S. Brunava L. (2016). Oat grain composition and its nutrition benefice. Agric. Agric. Sci. Proc. 8, 252–256. doi: 10.1016/j.aaspro.2016.02.100

[B47] Sun W. N. Gao X. M. Wang W. Wen L. Chao K. T. Gao Q. H. . (2014). Correlation analysis between agronomic traits and yield of oat. Inner Mongolia Agric. Sci. Technol. 06), 19–43. doi: 1007—0907(2014)06—0019—01

[B48] Van Zanten H. H. E. Herrero M. Van Hal O. Röös E. Muller A. Garnett T. . (2018). Defining a land boundary for sustainable livestock consumption. Global Change Biol. 24, 4185–4194. doi: 10.1111/gcb.14321, PMID: 29788551

[B49] Varga M. Jójárt R. Fónad P. Mihály R. Palágyi A. (2018). Phenolic composition and antioxidant activity of colored oats. Food Chem. 268, 153–161. doi: 10.1016/j.foodchem.2018.06.035, PMID: 30064743

[B50] Veda D. J. S. Chakraborti P. (2020). Application of priming through organic compounds in oat (Avena sativa L.) seed production. Int. J. Chem. Stud. 8, 2549–2553. doi: 10.22271/chemi.2020.v8.i2am.9131

[B51] Wang Q. Ellis P. R. (2014). Oat β-glucan: physico-chemical characteristics in relation to its blood-glucose and cholesterol-lowering properties. Brit. J. Nutr. 11, 4–13. doi: 10.1017/S0007114514002256, PMID: 25267243

[B52] Wang X. He Y. Zha Y. Chen H. Wang Y. Wu X. . (2024). Mapping winter fallow arable lands in Southern China by using a multi-temporal overlapped area minimization threshold method. GIScience Remote Sens. 61, 2333587. doi: 10.1080/15481603.2024.2333587

[B53] Wang J. Y. Liu Q. Sun Q. Z. Hao H. Liu H. S. T. Qiao X. F. . (2020). Study on production performance of 10 oat varieties in Wumeng mountain area. Agrostology 01), 49–55. doi: 10 3969/ j issn 2096-3971 2020 01 008

[B54] Wang J. L. Wang X. J. Liu Q. L. Liang G. L. Jv Z. L. Shi H. M. . (2024). Comprehensive evaluation of production performance and nutritional quality of different oat varieties in Sanjiangyuan area. J. Agric. Sci. 10), 1–13. doi: 10.11686/cyxb2024038

[B55] Xu T. M. Tian B. Q. Sun Z. D. Xie B. J. (2011). Changes in the three major nutrients during oat germination. Natural Product Res. Dev. 23, 534–537. doi: 10.16333/j.1001-6880.2011.03.032

[B56] Yan Y. D. Nie Y. Y. Xu L. J. Gao X. F. Rao Y. Z. Rao X. (2023). Evaluation of the potential of functional oat varieties in winter fallow fields in the southwestern mountainous region. J. Grassland 32, 42–53. doi: 10.11733/j.issn.1007-0435.2023.04.016

[B57] Yang M. Xu S. H. Rao X. Gao X. Xue W. Wu X. J. . (2023). Evaluation of agronomic traits and nutritional quality of forage oats in the Wumeng mountain cold area. Acta Agrestia Sin. 31, 1071–1080. doi: 10.11686/cyxb2022330

[B58] Zhang G. Y. Ma H. P. Shao X. M. Wang J. W. Shen Z. X. Fu G. (2019). Comparative study on the production performance and nutritional quality of 9 introduced oat varieties in Tibetan River Valley region. Acta Prataculturae Sin. 28, 121–131. doi: 10.11686/cyxb2018300

[B59] Zhao X. N. Gao Z. H. Wang B. Zhang Y. Y. Hu H. Y. Lan J. . (2023). Comparison and evaluation of drought resistance of 15 forage oat germplasm materials at seedling stage. J. Grassland Sci. 31, 3734–3743. doi: 10.11733/j.issn.1007-0435.2023.12.018

[B60] Zhao Y. H. Wang Z. L. Du J. C. Liu J. Y. Li Ji H. (2018). Comparative study on the introduction of oat germplasm resources and main agronomic traits. J. Northern Agric. Sci. 46, 1–9. doi: 10.3969/j.issn.2096-1197.2018.02.01

[B61] Zhou X. Lin W. Tong L. Liu X. Zhong K. Liu L. . (2016). Hypolipidaemic effects of oat flakes and β-glucans derived from four Chinese naked oat (Avena nuda) cultivars in Wistar-Lewis rats. J. Sci. Food Agric. 96, 644–649. doi: 10.1002/jsfa.7135, PMID: 25683724

